# Morphologic outcome of bimaxillary surgery–An anthropometric appraisal

**DOI:** 10.4317/medoral.19978

**Published:** 2014-12-05

**Authors:** Gregor F. Raschke, Ulrich M. Rieger, Andre Peisker, Gabriel Djedovic, Marta Gomez-Dammeier, Arndt Guentsch, Oliver Schaefer, Stefan Schultze-Mosgau

**Affiliations:** 1 MD, DMD, PhD, Department of Oral & Maxillofacial Surgery / Plastic Surgery, Friedrich Schiller University Jena, Erlanger Allee 101, 07747 Jena, Germany; 2 MD, PhD, Department of Plastic & Aesthetic, Reconstructive & Hand Surgery, St. Markus Hospital, Johann Wolfgang von Goethe University, Frankfurt/Main, Germany; 3 DMD, Department of Oral & Maxillofacial Surgery / Plastic Surgery, Friedrich Schiller University Jena, Erlanger Allee 101, 07747 Jena, Germany; 4 MD, Department of Plastic & Aesthetic, Reconstructive & Hand Surgery, St. Markus Hospital, Johann Wolfgang von Goethe University, Frankfurt/Main, Germany; 5 DMD, Department of Oral & Maxillofacial Surgery / Plastic Surgery, Friedrich Schiller University Jena, Erlanger Allee 101, 07747 Jena, Germany; 6 DMD, Interdisciplinary Research Groupe of Computational Medicine, Friedrich Schiller University Jena, Erlanger Allee 101, 07747 Jena, Germany; 7 MD, DDS, PhD, Department of Oral & Maxillofacial Surgery / Plastic Surgery, Friedrich Schiller University Jena, Erlanger Allee 101, 07747 Jena, Germany

## Abstract

Objectives: To adequately perform orthognathic surgery procedures, it is from basic interest to understand the morphologic changes caused by orthognathic surgery. Anthropometric analyses of standardized frontal view and profile photographs could help to investigate and understand such changes. Study Design: We present a pre- to postoperative evaluation of orthognathic surgery results based on anthropometric indices described by Farkas and cephalometric measurements. 30 Class III patients undergoing maxillary advancement by Le Fort I Osteotomy and mandibular setback by bilateral sagittal split osteotomy were evaluated. Preoperative as well as three and nine months postoperative lateral cephalograms as well as standardized frontal view and profile photographs were taken. On the photographs 21 anthropometric indices given by Farkas were evaluated. In cephalograms SNA and SNB angle as well as Wits appraisal were investigated. Results: The investigated anthropometric indices showed a significant increase of the vertical height of the upper lip without changing the relation of the upper vermilion to the cutaneous upper lip. The lower vermilion height increased relatively to the cutaneous lower lip without vertical changes in the lower lip. Due to maxillary advancement the upper face height increased meanwhile the lower face height decreased due to mandibular setback. SNA and SNB angle and Wits appraisal showed typical changes related to surgery. Conclusions: The investigated photo-assisted anthropometric measurements presented reproducible results related to bimaxillary surgery.

** Key words:**Orthognathic surgery, bimaxillary surgery, anthropometry, Class III.

## Introduction

Improvement of the occlusal function and acquisition of a harmonious and aesthetic appearance are major goals in orthognathic treatment and surgery (1).

Beside a throughout understanding of the underlying anatomy is a differentiated knowledge of orthognathic surgery related changes of the facial region fundamental in planning successful orthognathic treatment. While both, bony and soft tissue, undergo considerable changes in orthognathic surgery, the appraisal of aesthetic outcomes after orthognathic surgery particularly depends on the investigation of soft tissue changes (2).

These soft tissue changes may be detected by anthropometric indices described by Farkas (3). They are related to attractiveness (4,5) and have proven useful to objectifiable quantify pre- to postoperative changes in facial reconstructive (6), traumatologic (7) and aesthetic (8) surgery. Furthermore they are widely used in the field of orthodontics (9,10).

We feel that photo-assisted facial anthropometric measurements may help to adequately rate the effect of bimaxillary orthognathic surgery on the facial appearance.

In the presented study we investigated the effect of orthognathic surgery on the facial appearance in a group of 30 Class III patients undergoing bimaxillary surgery for maxillary advancement and mandibular setback. Preoperative anatomic landmarks and facial relationships were measured on standardized photographs. Changes resulting from surgery were measured three and nine months post operatively and compared to the preoperative values.

Currently, most studies report pre- to postoperative facial changes by cephalometric measurements on lateral cephalograms (11-13). Thus, cephalometric measurements of SNA and SNB angle as well as Wits appraisal were performed as well.

## Patient and Methods

All patients were operated at the Department of Oral & Maxillofacial Surgery at the University Hospital Jena, Germany. Before the study was initiated, the local Ethics Committee of the University Hospital Jena was asked to give his approval to the study. Because the study design aimed to evaluate routinely performed documentation like standardized photographies or X-rays and did not influence the the diagnostical or therapeutic process the Ethics Committee denied the necessity of special ethical approval. Prior to surgery all included patients signed an informed consent permitting the scientific evaluation of their routinely recorded documentation including x-rays and photographies.

All operations were performed in a standardized manner. All patients underwent orthodontics and orthognathic surgery, but no genioplasty or rhinoplasty and all patients exhibited a bilateral dentition of at least first molar to first molar. Patients with congenital deformities, such as cleft lip and/or palate, were excluded.

Maxillary advancement was in the known standardized manner performed via Le Fort I Osteotomy. Mandibular setback by bilateral sagittal split osteotomy was performed in the earlier described manner, too (14,15).

**Figure 1:**
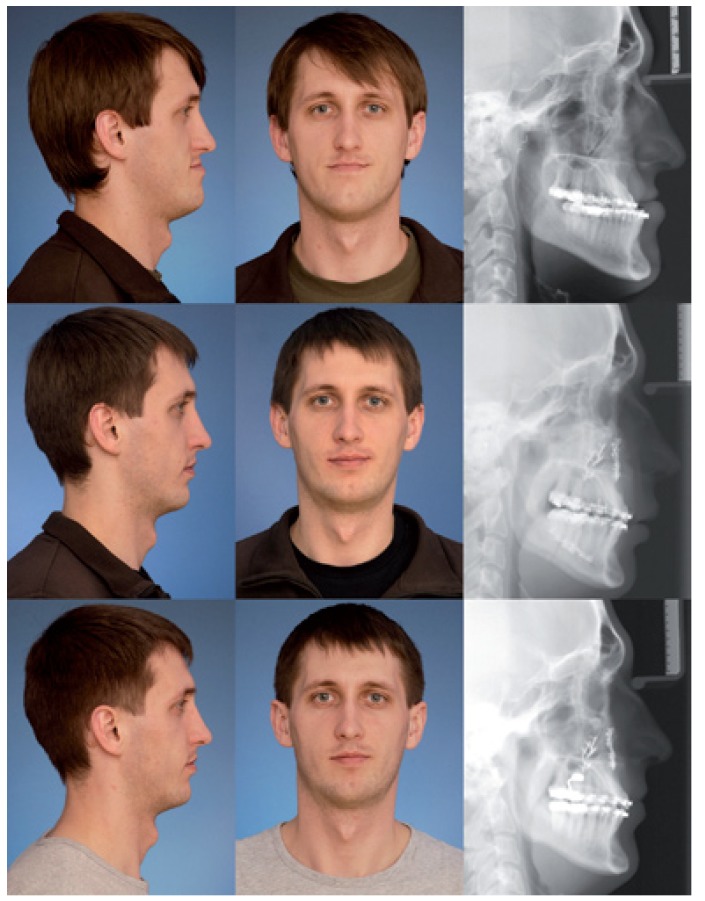
Standardized photographs and lateral cephalograms of a 25 year-old man undergoing bimaxillary surgery. Preoperative situation above, three months postoperative in the middle and nine months postoperative below.

A photographic and cephalometric description of an exemplary patient is shown in figure 1.

-Objective Rating Scheme

Coloured frontal view and profile photographs were taken the day before surgery. Postoperative photographs were taken three and nine months later with a Nikon D 80 camera (objective: Nikon AF Micro Nikkor 105 mm 1:2.8 D; aperture: f13; Nikon Corp, Tokyo, Japan) in a standardized manner as described elsewhere (16). All photographs were taken by a professional photographer. Analysis was performed using the Adobe Photo shop CS2 (Adobe Inc, San Jose, CA) software tool.

**Figure 2:**
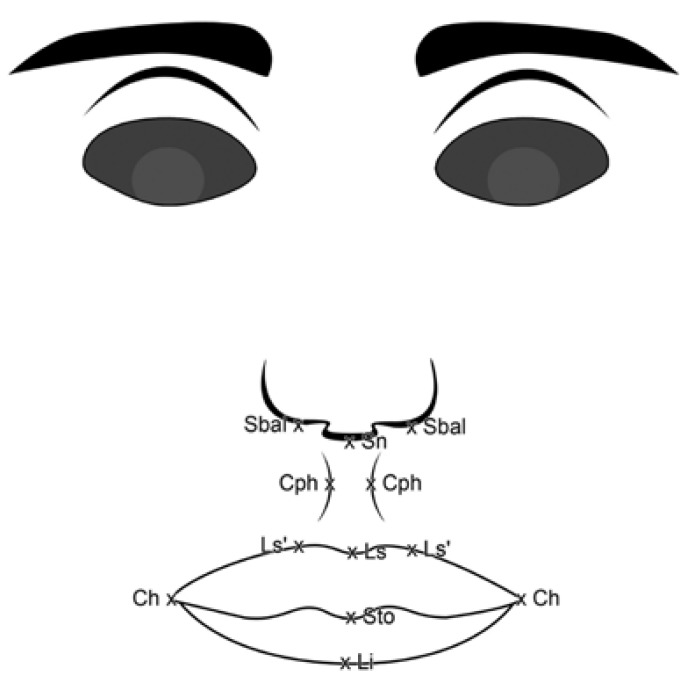
Schematic frontal-view image with description of the used anthropometric distances. Mouth width (ch-ch), philtrum width (cph-cph), upper lip height (Ls-Sn), lateral upper lip height (sbal-ls´), upper vermilion arc (ch-ls-ch), and lower vermilion arc (ch-li-ch).

Based on anthropometric values described by Farkas (17-19) predefined anatomic landmarks ( Table 1) and distances ( Table 2) were used to calculate the following indices ( Table 3) in the frontal view photographs (Fig. 2): (1) Upper lip height-mouth width index, representing the vertical distance between the subnasale and the stomion (ULH, sn-sto) as percentage of the mouth width (MW, ch-ch).) (2). Philtrum mouth width index, the philtrum width between the two crista philter (PW, cph-cph), as percentage of the mouth width between the two cheilions (MW, ch-ch) (3). Medial-lateral cutaneous upper lip height index representing the cutaneous upper lip height, the vertical distance between the labiale superius and the subnasale (CULH, sn-ls), as percentage of the lateral upper lip height, the vertical distance between the subalare and the lateral labiale superius beyond the subalare (LULH, sbal-ls´) (4). Upper vermilion contour index, the mouth width (MW) as percentage of the upper vermilion arc (UVA, ch-ls-ch) (5). Lower vermilion contour index, the mouth width (MW) as percentage of the lower vermilion arc (LVA, ch-li-ch) (6). Vermilion arc index, the lower vermilion arc (LVA) as percentage of the upper vermilion arc (UVA).

**Table 1:**
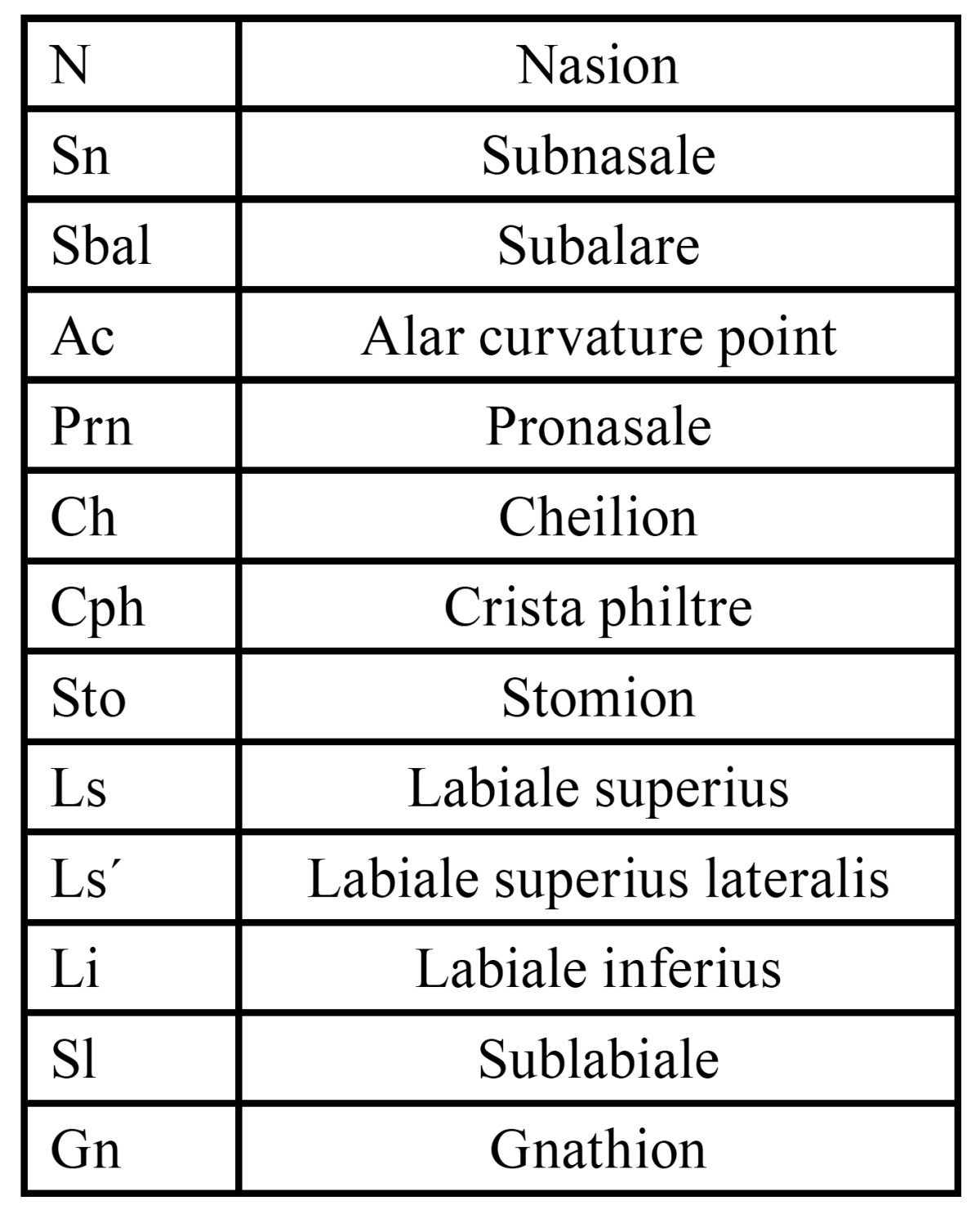
Used anthropometric landmarks based on the investigations by Farkas and Munro.

**Table 2:**
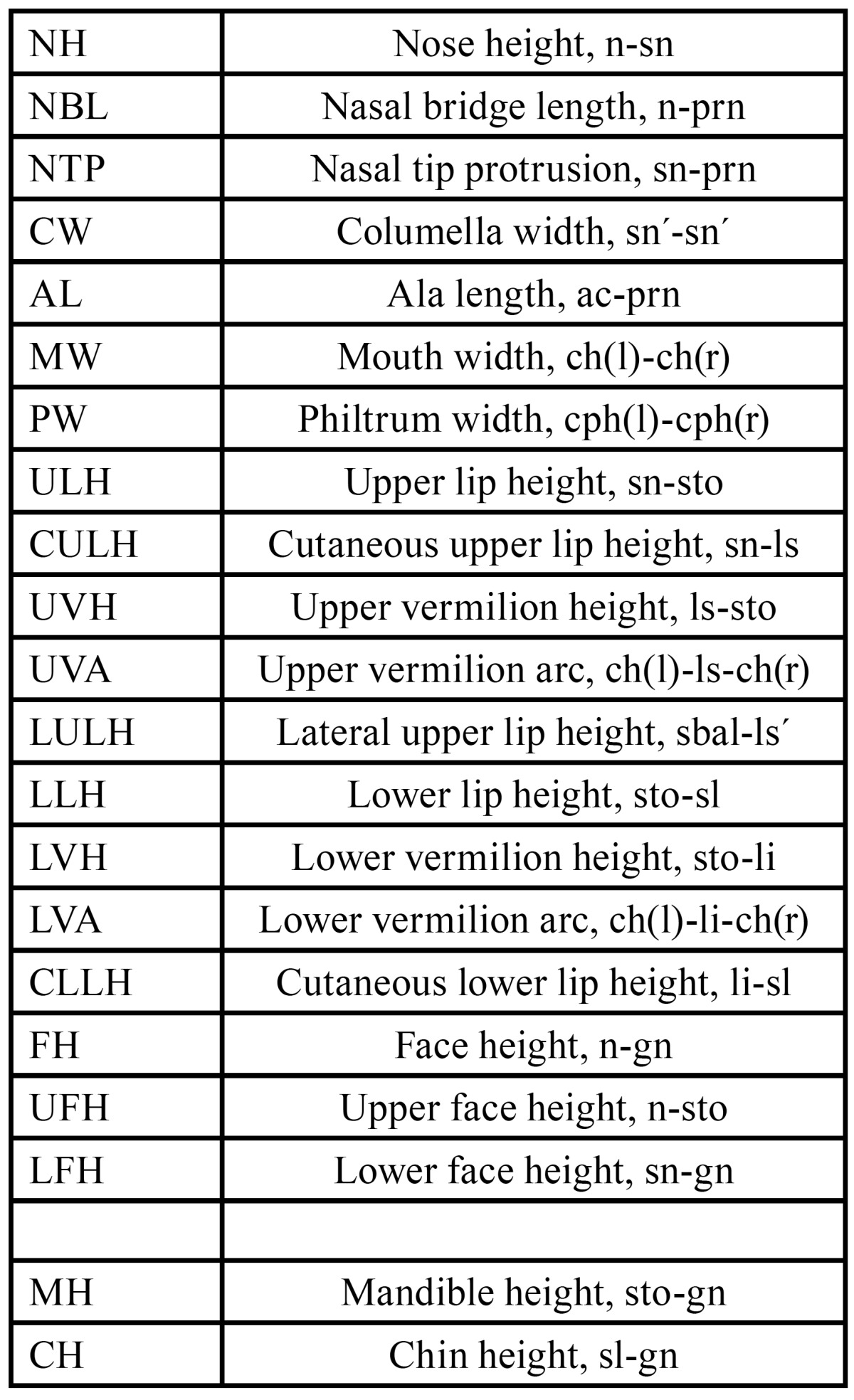
Used anthropometric distances based on the investigations by Farkas and Munro.

**Table 3:**
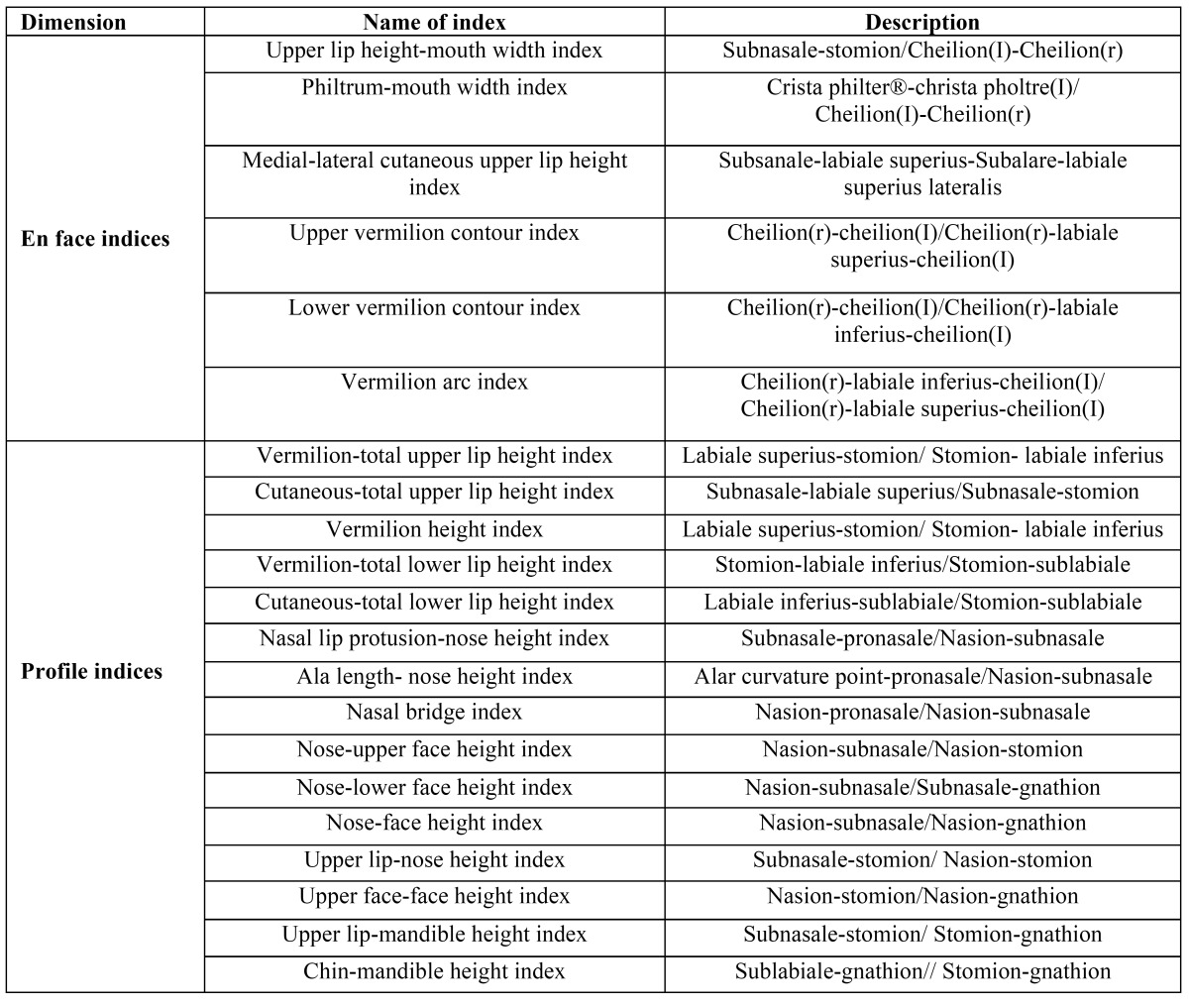
Used anthropometric indices based on the investigations by Farkas and Munro.

**Figure 3:**
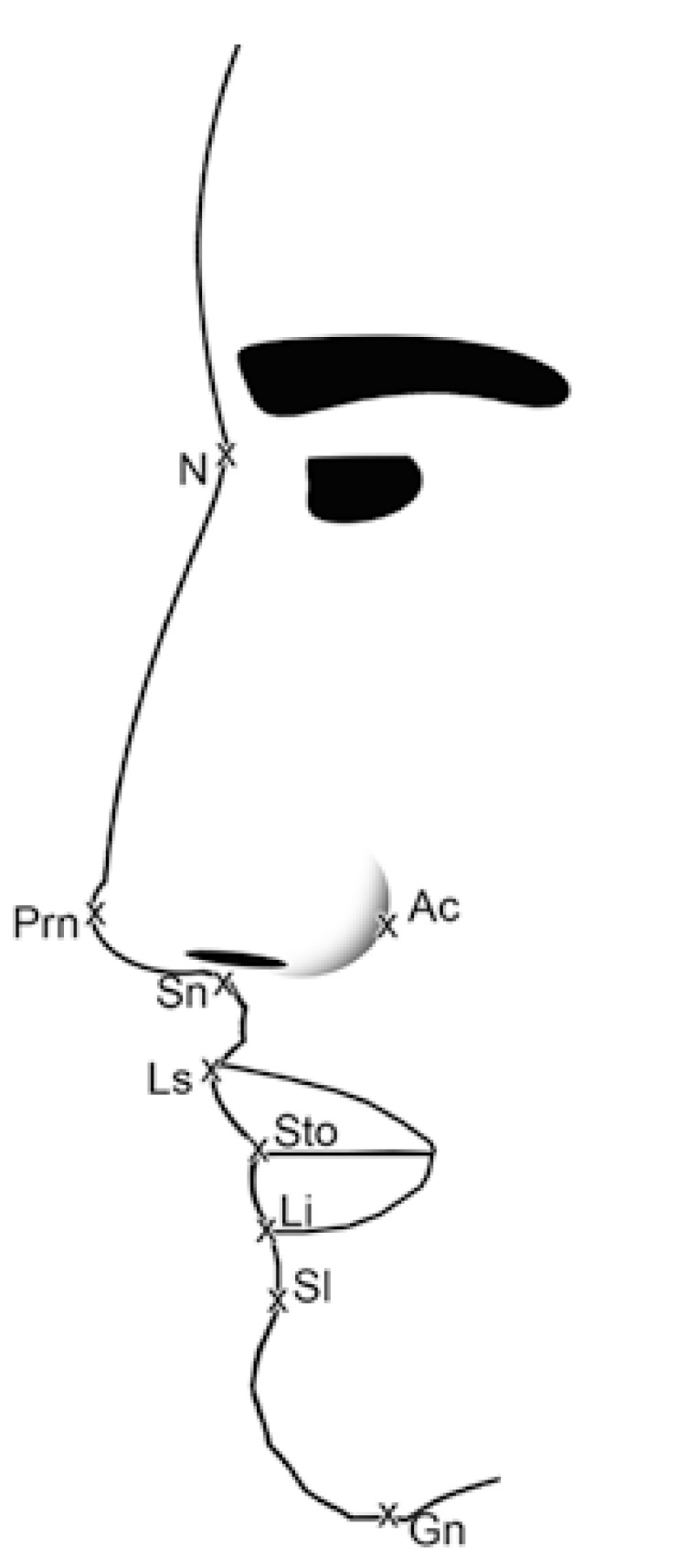
Schematic profile-view image with description of the used anthropometric distances. Nose height, N-Sn, Nasal tip protrusion, Sn-Prn, Ala length, Ac-Prn, Upper vermilion height, Ls-Sto, lower vermilion height , Sto-Li, cutaneous upper lip height , Sn-Ls, cutaneous lower lip height, Li-Sl, upper vermilion height, Ls-Sto, lower vermilion height, Sto-Li, total upper lip height, Ls-Sto, total lower lip height, Sto-Sl, face height, N-Gn, upper face height, N-Sto, lower face height, Sn-Gn, mandible height, Sto-Gn, chin height, Sl-Gn.

In the profile photographs the following data were recorded (Fig. 3): (1) Vermilion total upper lip height index represented by the upper vermilion height, the vertical distance between labial superius and stomion (UVH, ls-sto), as percentage of the upper lip height (ULH, sn-sto) (2). Cutaneous total upper lip height index, the vertical distance between cutaneous upper lip height (CULH, sn-ls) as percentage of the upper lip height, the vertical distance between subnasale and stomion (ULH, sn-sto) (3). Vermilion height index, represented by the upper vermilion height (UVH, ls-sto), as percentage of the lower vermilion height (LVH, sto-li) (4). Vermilion total lower lip height index, the lower vermilion height, the vertical distance between stomion and labiale inferius (LVH, sto-li) as percentage of the lower lip height (LLH, sto-sl) (5). Cutaneous total lower lip height index represented by the cutaneous lower lip height, the vertical distance between the labiale inferius and the sublabiale (CLLH, li-sl), as percentage of the lower lip height, the vertical distance between the stomion and the sublabiale (LLH, sto-sl) (6). Nasal tip protrusion-nose height index, the nasal tip protrusion (NTP, sn-prn), as percentage of the nose height (NH, n-sn) (7). Ala length-nose height index, representing the ala length (AL, ac-prn), as percentage of the nose height (NH, n-sn) (8) Nasal bridge index, the nasal bridge length (n-prn) as percentage of the nose height (n-sn) (9). Nose- upper face height index, the nose height (NH, n-sn), as percentage of the upper face height (UFH, n-sto) (10). Nose- lower face height index, the nose height (NH, n-sn), as percentage of the lower face height (LFH, sn-gn) (11). Nose- face height index, the nose height (NH, n-sn), as percentage of the face height (FH, n-gn) (12). Upper lip nose height index, the upper lip height (ULH, sn-sto), as percentage of the nose height (NH, n-sn) (13). Upper face- face height index, the upper face height (UFH, n-sto), as percentage of the face height (FH, n-gn) (14). Upper lip- mandible height index, representing the upper lip height (ULH, sn-sto), as percentage of the mandible height (MH, sto-gn) (15). Chin- mandible height index, the chin height (CH, sl-gn), as percentage of the mandible height (MH, sto-gn).

Lateral cephalograms were taken preoperatively as well as three and nine months post operatively. SNA and SNB angle as well as Wits appraisal as established cephalometric measurements in the appraisal of orthognathic surgery were raised.

-Statistical Analysis

An univariate ANOVA was conducted to evaluate effects of time (preoperative, three and nine months postoperative) on all variables. In case of a significant effect of time for a variable, post hoc comparisons with Bonferroni correction were applied.

## Results

All 30 white Caucasian Class III patients, 19 (63.3%) men and 11 (36.7%) women included in this study underwent maxillary advancement and mandibular setback. Average age was 32.11±10.47 years at time of surgery.

Results of the cephalometric and photographic measurements are shown in  Table 4.

**Table 4:**
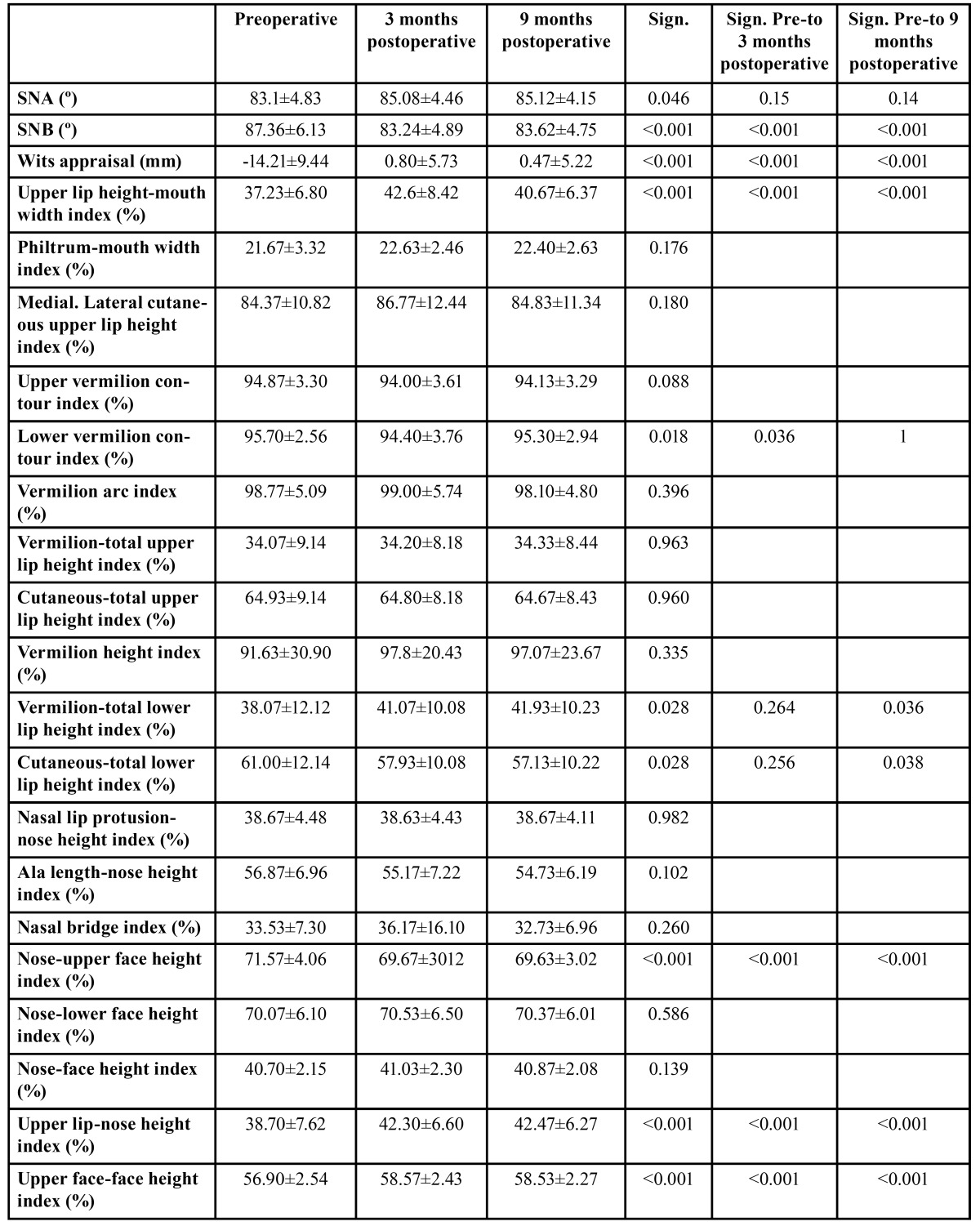
Comparison of pre- to postoperative cephalometric and anthropometric measurements.

SNB angle (*p*<.001) and Wits appraisal (*p*<.001) presented significant changes in the comparison of pre- to postoperative values three as well as nine months after surgery. SNA angle significantly changed pre- to postoperatively, too (*p*=.046). After Bonferoni correction this effect was not exactly allocatable. Anyhow, statistical analysis allows to interpret at least the existence of a significant effect of time.

The photo-assisted anthropometric measurements of upper lip height-mouth width index (*p*<.001), lower vermilion contour index (*p*=.036), nose-upper face height index (*p*=.001), upper lip-nose height index (*p*<.001), upper face-face height index (*p*<.001), upper lip-mandible height index (*p*<.001) and chin-mandible height index (*p*<.001) yielded significant pre- to postoperative changes three months after surgery.

Nine months after surgery upper lip height-mouth width index (*p*=.001), vermilion-total lower lip height index (*p*=.036), cutaneous-total lower lip height index (*p*=.038), nose-upper face height index (*p*<.001), upper lip-nose height index (*p*<.001), upper face-face height index (*p*<.001), upper lip-mandible height index (*p*<.001) and chin-mandible height index (*p*<.001) yielded significant changes as compared to the preoperative values.

## Discussion

-Discussion of the Method

The desire to improve facial aesthetic and appearance is an important factor in seeking orthognathic treatment (20,21).

A number of increasingly sophisticated techniques are available for orthognathic treatment and surgery planning (1). Currently, the most used method to analyses pre- to postoperative changes of hard and soft tissue is two dimensional analysis by cephalograms (22,23). Three dimensional models based on various techniques (2,11,22,24) are also in use, but because of high costs and difficult application not clinical routine.

In an earlier study we showed the value of photo-assisted anthropometric measurements to get a deeper understanding of facial morphologic changes related to mandibular advancement in Class II patients (3). In the presented study we investigated bimaxillary surgery related changes on the facial morphology of Class III patients.

The 21 anthropometric indices ( Table 3) presented here were selected because of the reliable exact identification of their corresponding anthropometric landmarks ( Table 1 1, Table 2) and their potential impact by bimaxillary surgery (19,20). To adequately evaluate facial pre- to postoperative changes, indices in profile as well as frontal view were investigated (12). In the following we describe the meaning of different facial aesthetic units and the investigated anthropometric landmarks and indices in bimaxillary orthognathic surgery:

Positioned in the center if the face, considerations about the morphology of the nose and its relation to upper lip and lower face are of major interest for aesthetic considerations in bimaxillary surgery. Nasion and subnasale are fundamental reference points in orthodontics and aesthetic surgery (5).

Located in the center of the face and dividing the upper lip in two lateral and one medial aesthetic subunits, the philtrum is of great importance for the facial appearance. Philtrum-mouth width index reflects the relation of philtrum and mouth width. Upper lip height-mouth width index describes the vertical extension of the upper lip to the horizontal extension of the mouth width. Together with the medial lateral cutaneous upper lip height index it reflects the relation of mouth width, upper lip, and nose to each other.

Upper and lower vermilion, their relation to each other and the upper and lower lips are from major importance for facial aesthetics. Their composition is directly influenceable by bimaxillary surgery. The vermilion-total upper and lower lip height indices describe the relation of the vermilions to the overall vertical height of their belonging lips. The vertical relation of the cutaneous fraction of the lips to the overall height of the lips describe the cutaneous-total upper and lower-lip height indices. Maxillary advancement and mandibular setback may have bigger impact on the vertical relations of upper and lower face, nose, mandible and chin. Considerations about the vertical relations of upper and lower face are not only beneficial in the planning and evaluation of bimaxillary surgery.In order to adequately rate the results of the anthropometric measurements, SNA and SNB angle as well as Wits appraisal as established cephalometric measurements in the estimation of orthognathic surgery were investigated as well (25,26).

Concerning the individual specifics of each patient, data of our patients were not differentiated between males and females as we did not aim at inter-individual changes or correlations. Instead pre- to postoperative changes were analyzed.

-Discussion of the Results

In the anthropometric measurements the significant increases of upper lip-mouth width index and upper lip nose height index pre- to post operatively indicate an increased visible vertical length of the upper lip due to maxillary advancement and mandibular setback.

Vermilion- and cutaneous-total upper lip height index did not show significant changes pre- to post operatively. This finding is an indicator, that the vertical relation of vermilion and cutaneous fraction of the upper lip was not influenced, meanwhile the total vertical upper lip length increased, which is a typical result after bimaxillary correction of Class III deformities (27). The anthropometric measurements presented by Farkas may help to precisely detect these dimensions regarding vermilion and cutaneous part of the upper lip.

In contrast to the upper lips, the significant increase of the vermilion-total lower lip height index and decrease of the cutaneous-total lower lip height index indicate a changed vertical relation between cutaneous fraction and vermilion of the lower lips in favor of the lower vermilion pre- to post operatively.

Mouth and philtrum width were reported earlier to present constant pre- and postoperative values after bimaxillary surgery in Class III patients (2). The constant pre- and postoperative values of the philtrum-mouth width index confirm this finding.

The significant increase of upper face-face height index, upper lip-mandible height index and chin-mandible height index as well as decrease of nose-upper face height index reflect the vertical shortening of the lower face due to mandibular setback and vertical lengthening of the upper face due to maxillary advancement (28). Although the post operatively increased chin mandible height index indicates an increased vertical chin to mandible height, often a weak chin with little prominence may result after bimaxillary surgery or isolated mandibular setback in Class III patients. In this case genioplasty increasing the submental length and chin prominence may be performed (29).

In the cephalometric measurements the preoperative means of SNA and SNB angle were similar to those reported in class III patients (30). The significant increase of SNA and decrease of SNB angle are typical results of bimaxillary correction of Class III malocclusion (31).
